# The impact of aging and thickness on flexural strength of various zirconia ceramics

**DOI:** 10.1186/s12903-024-04745-1

**Published:** 2024-08-20

**Authors:** Oyku Silmeoglu Yagli, Esra Talay Cevlik, Duygu Kurklu Arpacay

**Affiliations:** 1 Faculty of Dentistry, Department of Prosthodontics, Aydın Adnan Menderes University, Efeler, Aydın, Türkiye; 2 Faculty of Dentistry, Department of Prosthodontics, Izmir Democracy University, Konak, Izmir, Türkiye

**Keywords:** Degradation, Strength, Thickness, Yttria-stabilized tetragonal zirconia, Zirconia ceramics

## Abstract

**Background:**

Effects of the aging process on the flexural strength of Y-TZP and different Y-PSZ ceramics of different thicknesses were investigated.

**Methods:**

300 disc-shaped samples (12 mm diameter, 0.8 and 1.5 mm thicknesses) were made from 5 different zirconia materials 3Y-TZP LA, 4Y-PSZ, 5Y-PSZ, 3 + 5Y-PSZ and 4 + 5Y-PSZ. Experimental groups were artificially aged in an autoclave at 134 °C, 2 bar pressure for 1 and 5 h; control groups were not subjected to any treatment. Microstructural analysis was conducted using Scanning Electron Microscopy, and X-Ray Diffraction analysis determined the crystalline phase content. The impact of aging on flexural strength was investigated with the use of the biaxial flexural strength test. Data were analyzed using three-way ANOVA tests with a significance level of *p* < 0.05, applying Bonferroni correction for multiple comparisons.

**Results:**

Statistically significant differences in flexural strength were observed among the materials and the material thicknesses (*p* < 0.05), while there were no significant differences among the aging times (*p* > 0.05). The highest mean flexural strength values were recorded in the case of the 3 Y-TZP-1.5 mm–5 h group (744.1 ± 61.2 MPa), which was attributed to phase-transformation toughening. The lowest values were observed in the case of the 5 Y-PSZ-1.5 mm–5 h (338.3 ± 34.8 MPa) group.

**Conclusions:**

Both material type and thickness significantly affect the flexural strength of zirconia ceramics, whereas aging time does not; thus, material selection and thickness are crucial considerations for clinicians.

## Background

Yttria-stabilized zirconia polycrystalline (Y-TZP) ceramics have been widely used in dental restorations due to their excellent mechanical properties and biocompatibility. The first generation of this material, known as ‘3Y-TZP’, contains 3 mol% yttria and 0.25% by weight alumina (Al_2_O_3_) as a stabilizer. This composition ensures stability in the tetragonal phase at room temperature and results in an opaque white structure. To enhance the aesthetic properties of 3Y-TZP, the alumina content can be reduced to 0.05%, creating second-generation zirconia known as ‘3Y-TZP LA’ (Low Alumina) [1]. Both generations of zirconia are noted for their superior flexural strength and high fracture toughness [2] attributed to their small-scale grain size (~ 0.3–0.6 μm) [3–5] and transformation toughening [6]. This toughening mechanism involves the metastable tetragonal grains transforming into monoclinic grains under stress, causing a 3–5% volume increase (martensitic transformation) that induces compressive stresses at crack tips and halts crack propagation [1, 2, 7]. Subsequent developments involving raising the yttria content to 5 mol% [2, 6], produced the third generation yttria-partially stabilized zirconia ‘5Y-PSZ’ while raising the yttria content to 4 mol% [2], created the fourth generation ‘4Y-PSZ’ [2]. This creates an additional cubic phase [2, 6] which is incapable of undergoing phase transformation [4]. The third and fourth generation zirconia exhibit less or no phase change [3, 4, 6] and the flexural strength of PSZ variants varies from 700 to 800 MPa and 600 to 900 MPa, respectively [8].

The aesthetic properties of monolithic Y-TZP restorations have been extensively studied. The absence of an amorphous glass phase in zirconia reduces its translucency and enhances its opacity [9]. To address this, multilayered materials with color- and translucency gradients have been developed, mimicking the natural appearance of teeth in monolithic restorations [10]. In these systems, opacity is higher in the gingival region, while translucency increases towards the incisal region, achieving a natural tooth-like appearance. Advanced monolithic color and strength-gradient zirconia combine the benefits of various zirconia generations, featuring a basal layer with improved mechanical properties (3Y-TZP or 4Y-PSZ) and a more translucent incisal layer (5Y-PSZ) [11].

However, monolithic zirconia restorations are susceptible to low-temperature degradation (LTD) in moist environments, where exposure to water induces a slow transition from the tetragonal to the monoclinic phase [12, 13]. The layered manufacturing process of zirconia blanks may also negatively impact mechanical properties [10]. It has been indicated that the impact of low-temperature degradation is more pronounced with decreasing material thickness [14]. Although research has predominantly focused on the translucency and fracture strength of new generation zirconia [5, 15], there is a paucity in vitro studies examining their mechanical properties compared with traditional zirconia, and even fewer investigating how stabilizer content affects phase transformation and mechanical properties.

The purpose of this study was to investigate the effects of aging duration (1 h and 5 h) on the flexural strength of different generations of zirconia ceramics of varying thicknesses (0.8 and 1.5 mm). Our hypothesis for the study is: There is no statistically significant difference in the flexural strength values of different zirconia materials, regardless of material thickness or aging time.

## Methods

The minimum sample size was calculated using data from previous research (the TP group with the lowest η^2^ value was selected in the study’s results section) [16] in the G*Power v.3.1.9.2 program (Heinrich Heine, University of Dusseldorf, Dusseldorf, Germany). To calculate the flexural strength differences between the study groups, an alpha error of 0.05 and a power of 0.95 were established, resulting in a minimum estimated sample size of 7 for each group. To enhance the statistical power of the study and account for potential sample loss, 10 samples per group were included. Consequently, the study comprised a total of 300 specimens.

**Fig. 1:**
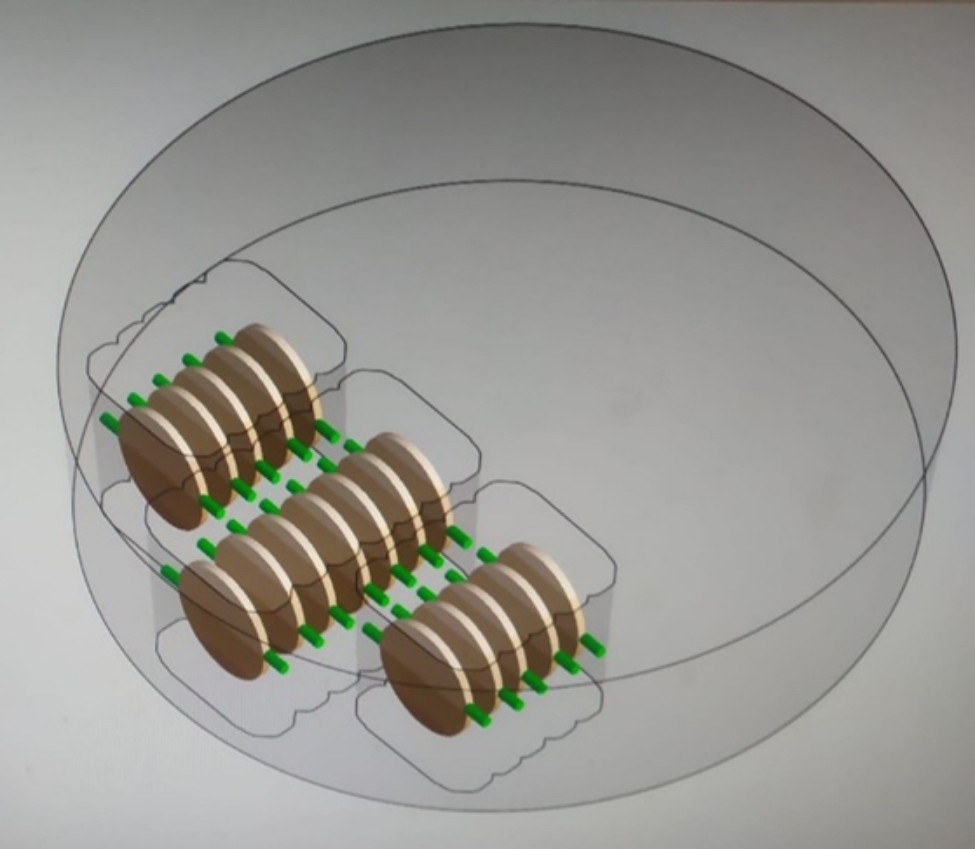
The design of the zirconia ceramic discs

The disc-shaped specimens (final dimensions: 12 mm in diameter, 0.8 and 1.5 mm thickness) were designed to be positioned in the center of the zirconia ceramics, with the long axis of the discs parallel to the short axis of the zirconia ceramic discs (Fig. 1). Pre-sintered zirconia ceramic discs with a diameter of 98.5 mm and a thickness of 18 to 20 mm, selected in accordance with the specimen sizes, were placed in the milling unit. A total of 300 specimens were fabricated from five different zirconia ceramics: 3Y-TZP LA (DD Bio ZX^2^ Color, Dental Direct, Germany), 4Y-PSZ (DD Cube One ML, Dental Direct, Germany), 5Y-PSZ (DD Cube X^2^ ML, Dental Direct, Germany) and color/strength-gradient zirconia materials 3 + 5Y-PSZ (IPS e. max ZirCAD Prime, Ivoclar Vivadent, Schaan, Liechtenstein), 4 + 5Y-PSZ (IPS e. max ZirCAD MT Multi, Ivoclar Vivadent, Schaan, Liechtenstein) by CAD/CAM (Dental Wings Software, DWOS, Turkuaz Dental, Izmir, Türkiye). Specimens were designed and milled in larger proportions to compensate for 20–25% shrinkage after sintering. After milling, the support points were cut to separate the specimens. Before sintering, the support point of the specimens were smoothened under running water using #600, #1000 and #1200 grit silicon carbide (SiC) papers (English Abrasives & Chemicals Ltd). All specimens were sintered as specified by the manufacturer, and the sintering process removed any monoclinic phase on the surface of the specimens during preparation [14].

Specimens of five different zirconia ceramics were divided into six subgroups, with 10 specimens in each test group. The test groups were labeled based on their generic names, thickness, and aging time. (DD Bio ZX^2^ Color: 3Y, DD Cube One ML: 4Y, DD Cube X^2^ ML: 5Y, IPS e. max ZirCAD Prime: 3 + 5Y, IPS e. max ZirCAD MT Multi: 4 + 5Y. For example: 3Y-0.8-1 indicates that DD Bio ZX^2^ Color with 0.8 mm thickness underwent 1 h of aging) The subgroups in each material that were not aged were designated as the control groups.

Specimens were artificially aged in an autoclave (HV-50, Upright Autoclave, Hirayama, Manufacturing Corp, Saitama, Japan) at 134 °C, under 2 bar pressure, for 1 h (*n* = 100) and 5 h (*n* = 100) according to ISO standards 13356. One hour of this ageing process at 134 °C is equivalent to 3–4 years of in vivo function [17], while control groups were not subjected to any aging condition. SEM analysis was performed to examine the surface topography of one randomly selected specimen from each subgroup. A total of 30 specimens were gold-coated using a gold plating device (Quorum Q150 ES, Quorum Technologies Ltd, East Sussex, United Kingdom). Prepared specimens were examined under SEM (Carl Zeiss, Sigma 300 VP, Jena, Germany) at magnifications of X1000, X5000, X10000 and X25000. Crystal structure analyses of the specimens were performed with an X-ray diffractometer (XRD) (Bruker D8 Advance, Bruker AXS GmbH, Karlsruhe, Germany) using a monochromatic CuKα beam. One specimen was randomly selected from each subgroups placed in the sample holder of the device. XRD scans were adjusted to 40 mA current and 40 kV voltage, and scanned between 20°- 40° 2θ angles with 0.019° step range. After the scans, diffraction graphs were produced and analyzed using computer software compatible with the system (EVA, Bruker AXS GmbH, Karlsruhe, Germany). For each specimen, the highest peak values in the regions where the density increased, and the diffraction angles at which these values were observed, were recorded. The monoclinic/tetragonal phase ratio (Xm) of the phase-changed monoclinic zirconia compared to the tetragonal phase was calculated using the equation below [18]. Relative intensities of the monoclinic (111), monoclinic (-111), and tetragonal phases are I_m_(111), I_m_(-111), and I_t_(111), respectively.

$$\:Xm=\frac{Im\left(111\right)+Im(-111)}{Im\left(111\right)+Im\left(-111\right)+It\left(101\right)}$$

The monoclinic volume content (Fm) was calculated using the formula below: [19]

$$\:Fm=\frac{1.311Xm}{1+0.311Xm}$$

The specimens underwent a biaxial flexural test in a universal testing machine (Marestek, Marestek, Mares Engineering, Istanbul, Türkiye). Each specimen was placed on three 3.2-mm diameter stainless steel balls, equidistantly placed from each other on a 10-mm diameter circle. The load was applied at the center of the specimens by a cylindrical-loading piston (1.4 mm diameter) until a fracture occurred at a cross-head speed of 1 mm/min according to ISO standards 6872. A computer attached to the test machine recorded the load at the time that specimens fractured. The formula below was used to convert the test results from N to MPa in accordance with international standards (ISO 6872).

S = − 0.2387 P(X- Y)/d^2^.

S: Maximum tensile stress (MPa),

P: Load causing fracture (N).

X = (1 + v) ln(r2/r3)^2^ + [(1-v)/2] (r2/r3)^2^.

Y = (1 + v) [1 + ln(r1/r3)^2^] + (1-v) (r1/r3)^2^.

(V): Poisson ratio (0.25).

r1: Radius of the support circle (mm),

r2: Radius of the loaded area (mm),

r3: Radius of the specimen (mm),

d: Thickness of the specimen at fracture origin (mm).

Data was analyzed using IBM SPSS Statistic V25.0 (SPSS Inc., Chicago, IL, USA). The Shapiro-Wilk test, kurtosis-skewness values, and histogram graphs were used to detect normal distribution; the Levene test was performed for homogeneity of variance. Data were analyzed using three-way Analysis of Variance (ANOVA) tests *p* < 0.05 set as significant, with Bonferroni correction for multiple comparisons. Descriptive analyses were used to present the results of the Xm and Fm results.

## Results

### SEM analysis

**Fig. 2:**
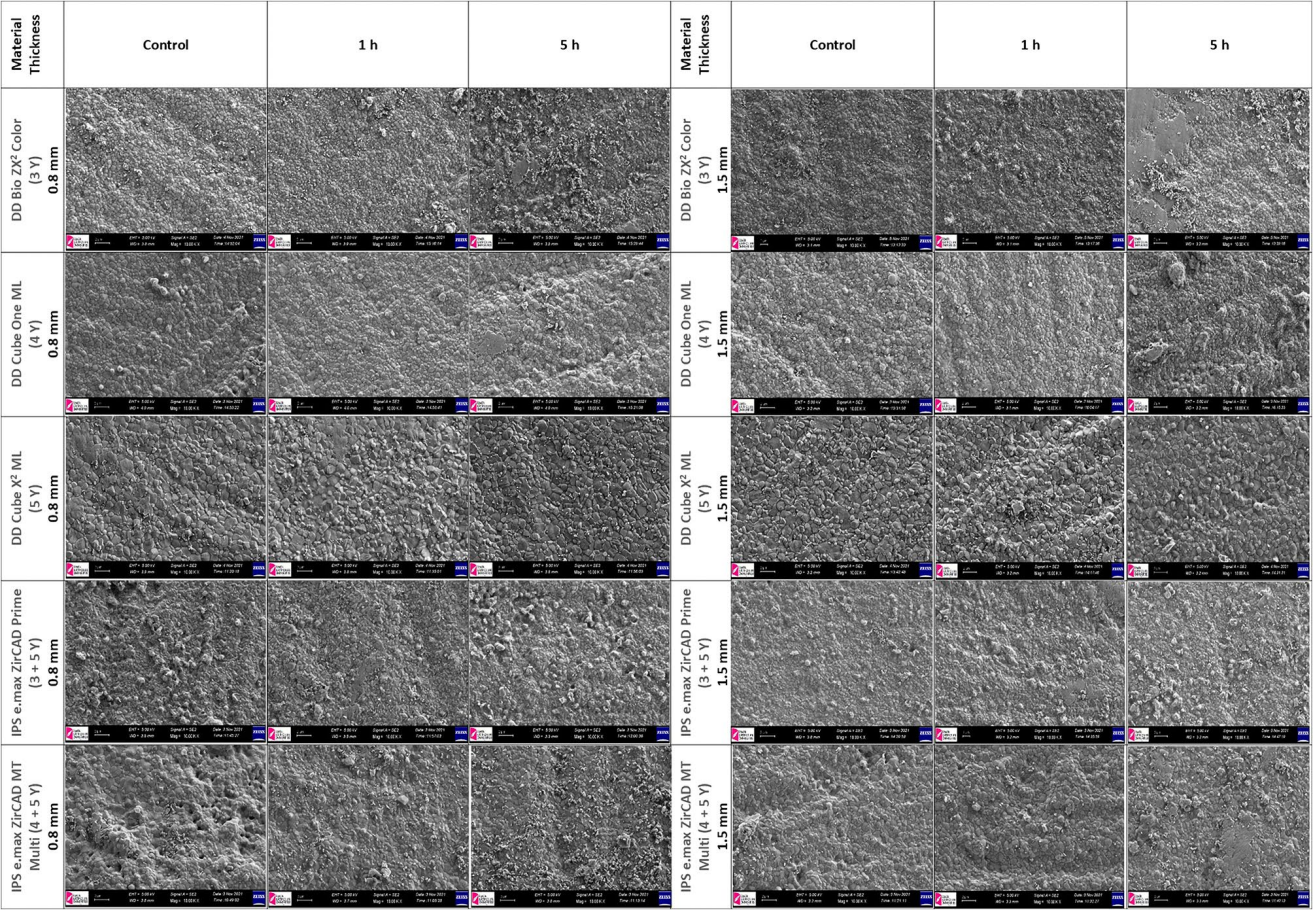
Scanning electron microscope images of the materials (magnification 10000 X)

SEM images of one representative specimen from each groups are shown in Fig. 2. The surface morphology, grain boundaries and continuity of the specimens were evaluated. When the SEM images were examined, deterioration in the surface morphology was observed after autoclave aging. Changes in topographic patterns caused by autoclave aging methods were found, and the textured surface appearance was noted on the specimen surfaces of 3Y-0.8-5 and 3Y-1.5-5.

### XRD analysis

**Fig. 3:**
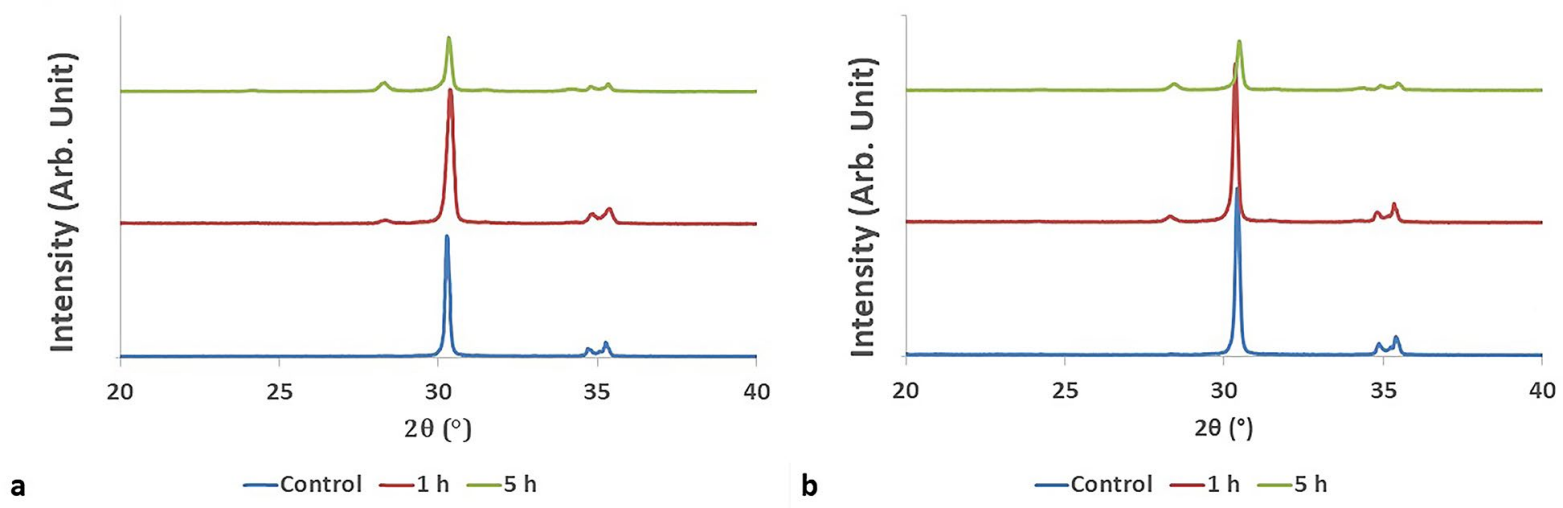
XRD graphs of a specimen taken from subgroups of 3 Y-TZP material

The Xm and Fm values are shown in Table 1. Depending on the aging time and thickness, the relative intensities of the tetragonal and monoclinic phases differed among the materials. The Fm values shows similarity to the Xm values. The Fm values of 3Y and (3 + 5)Y groups differs with increasing aging time. A representative XRD pattern of the specimens of 3Y group were given in Fig. 3(a-b), (a) represents the XRD graph of the 3Y-0.8 mm specimens, while (b) represents 3Y-1.5 mm specimen. According to the result of the XRD analysis, monoclinic and tetragonal peak intensities differ in terms of aging time. In the 3Y and (3 + 5)Y groups the monoclinic phase increased with the increase of the aging time.

**Table 1:** Xm and Fm values by groups (%)

	3 Y 0.8 mm		3 Y 1.5 mm		4 Y 0.8 mm		4 Y 1.5 mm		5 Y 0.8 mm	
	Xm	Fm	Xm	Fm	Xm	Fm	Xm	Fm	Xm	Fm
**Control**	0.05	0.07	0.03	0.04	0.04	0.05	0.03	0.04	0.04	0.06
**1 h**	0.07	0.09	0.07	0.09	0.03	0.04	0.03	0.05	0.04	0.06
**5 h**	0.20	0.20	0.17	0.21	0.06	0.08	0.06	0.08	0.05	0.06
	**5 Y** **1.5 mm**		**(3 + 5)Y** **0.8 mm**		**(3 + 5)Y** **1.5 mm**		**(4 + 5)Y** **0.8 mm**		**(4 + 5 )Y** **1.5 mm**	
	**Xm**	**Fm**	**Xm**	**Fm**	**Xm**	**Fm**	**Xm**	**Fm**	**Xm**	**Fm**
**Control**	0.04	0.06	0.04	0.05	0.06	0.07	0.03	0.04	0.06	0.08
**1 h**	0.05	0.06	0.08	0.10	0.10	0.13	0.04	0.06	0.04	0.05
**5 h**	0.05	0.06	0.18	0.22	0.13	0.16	0.03	0.04	0.05	0.06

### Biaxial flexural strength

**Fig. 4:**
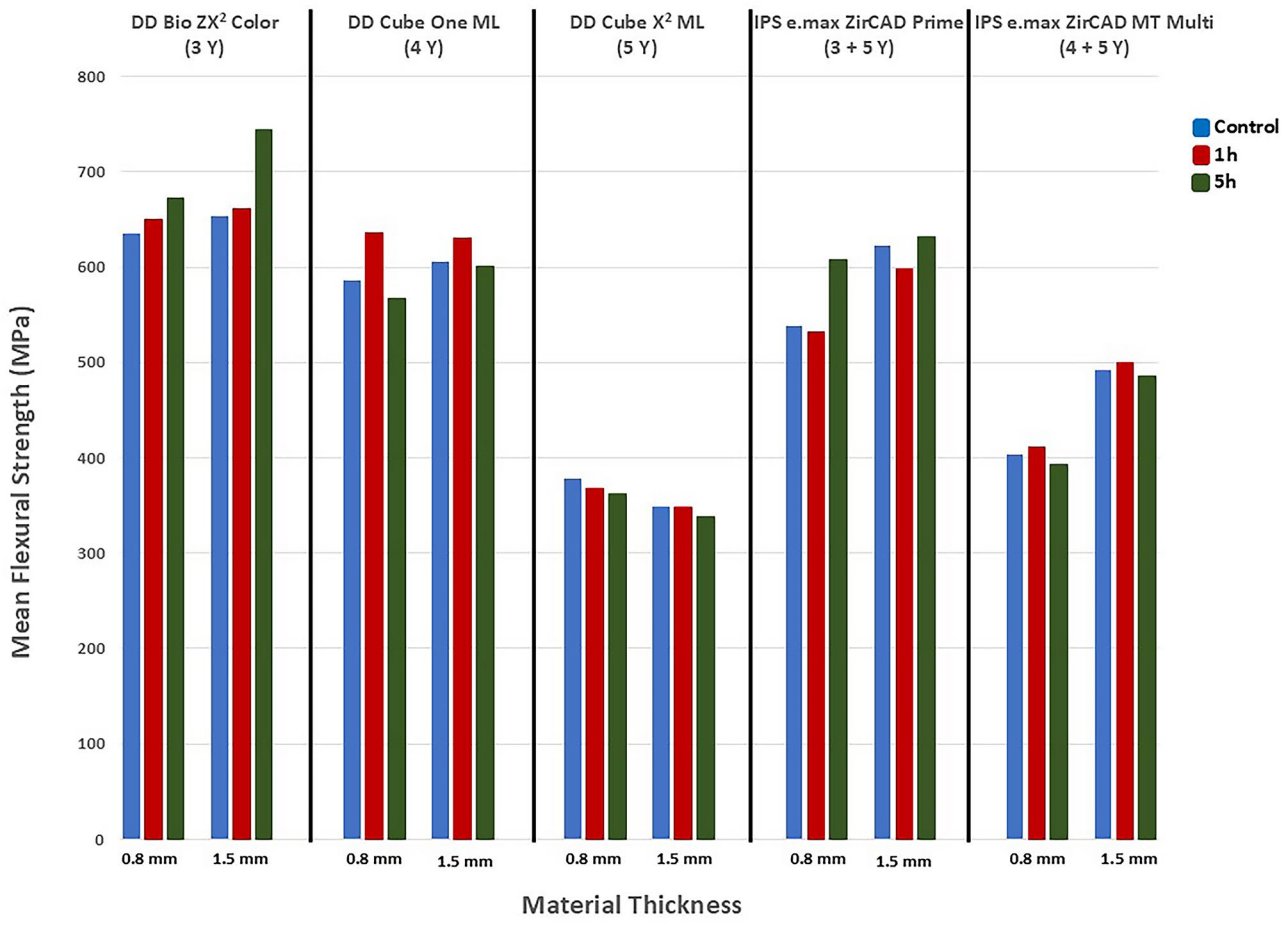
Flexural strength values

The mean biaxial flexural strength data are shown in Fig. 4. Based on three-way ANOVA results, there was a significant difference among the ceramics in terms of the mean flexural strength values (F = 187.907, *p* = 0.000, η^2^ = 0.736). Based on the Eta Square (η^2^) value, the impact of materials on flexural strength is great (η^2^ > 0.14).

There was a significant difference in the mean flexural strength values in terms of material thicknesses (F = 17.358, *p* = 0.000, η^2^ = 0.060) and, according to the Eta Square (η^2^) value, thickness has a moderate effect on the flexural strength of the materials.

No statistically significant difference was found in terms of the aging (F = 0.916, *p* > 0.05, η^2^ = 0.007). While the interaction of the independent variables in terms of material*thickness (F = 5.376, *p* = 0.000, η^2^ = 0.074) and material*aging (F = 2.381, *p* = 0.017, η^2^ = 0.066) have a moderate effect on the flexural strength, the interaction of thickness*aging (F = 0.176, *p* > 0.05, η^2^ = 0.001) and material*thickness*aging time (F = 0.551, *p* > 0.05, η^2^ = 0.016) had no effect on flexural strength.

The post hoc Bonferroni test indicated that the difference among the groups was statistically significant (*p* < 0.05) except between the 4Y and (3 + 5)Y groups (*p* ˃ 0.05) Table 2. Statistically, the highest mean flexural strength values were observed in the 3Y group, followed by the 4Y, (3 + 5)Y, (4 + 5)Y, and 5Y groups respectively, while no significant difference was found between the 4Y and (3 + 5)Y groups. The mean flexural strength value of the materials with 1.5 mm thickness was statistically higher than with regard to 0.8 mm thickness (*p* = 0.000).

**Table 2:** Pairwise comparison of the materials

Materials		Mean difference	Standard error	*p*	
**DD Bio ZX** ^**2**^ **Color** **(3Y)**	4Y	65.069	13.147	0.000*	
	5Y	312.121	13.147	0.000*	
	(3 + 5)Y	81.183	13.147	0.000*	
	(4 + 5)Y	222.068	13.147	0.000*	
**DD Cube One ML** **(4Y)**	3Y	-65.069	13.147	0.000*	
	5Y	247.052	13.147	0.000*	
	(3 + 5)Y	16.114	13.147	1.000	
	(4 + 5)Y	156.999	13.147	0.000*	
**DD Cube X** ^**2**^ **ML** **(5Y)**	3Y	-312.121	13.147	0.000*	
	4Y	-247.052	13.147	0.000*	
	(3 + 5)Y	-230.938	13.147	0.000*	
	(4 + 5)Y	-90.053	13.147	0.000*	
**IPS e.max ZirCAD Prime** **(3 + 5)Y**	3Y	-81.183	13.147	0.000*	
	4Y	-16.114	13.147	1.000	
	5Y	230.938	13.147	0.000*	
	(4 + 5)Y	140.885	13.147	0.000*	
**IPS e.max ZirCAD MT Multi** **(4 + 5)Y**	3Y	-222.068	13.147	0.000*	
	4Y	-156.999	13.147	0.000*	
	5Y	90.053	13.147	0.000*	
	(3 + 5)Y	-140.885	13.147	0.000*	

*P < 0.05

Three-way ANOVA followed by post hoc Bonferroni tests were conducted, and according to the results, the material*thickness interactions of (3 + 5)Y and (4 + 5)Y materials had statistically significant effects (*p* = 0.002, *p* = 0.000) in terms of flexural strength values (*p* < 0.05). In both materials, the flexural strength increased as the thickness increased. The material*thickness interactions of 3Y, 4Y and 5Y groups did not have a statistically significant effect on flexural strength (*p* > 0.05). Only in the case of the 3Y group did the material*aging interaction give statistically significant results in terms of flexural strength (*p* = 0.012). There was a statistically significant difference between the control and 5-hour aging time groups in terms of 3Y material (*p* = 0.016). The flexural strength value of the 3Y-control was less than of the 3Y-5 h aging group.

## Discussion

In this study, zirconia materials 3Y-TZP LA (DD Bio ZX^2^ Color), 4Y-PSZ (DD Cube One ML), 5Y-PSZ (DD Cube X^2^ ML) and color/strength-gradient zirconia materials 3 + 5Y-PSZ (IPS e. max ZirCAD Prime), 4 + 5Y-PSZ (IPS e. max ZirCAD MT Multi) were examined to compare their flexural strength. The results showed no significant interaction between material type, thickness and aging time. However, there are significant differences in the flexural strength values based on material type and thickness, while aging times did not have a significant effect. This results in partial acceptance of the null hypothesis.

Previous studies have used various specimen shapes in in vitro settings, such as disc-shaped [5, 7, 12, 20–22], rectangular, [20] and bar-shaped specimens [16, 23]. Disc-shaped specimens in particular have been produced with different diameters (12 mm [12], 14 mm [21], 15 mm [5, 7, 20]) and thicknesses (0.5 mm [5], 1 mm [20], 1.2 mm [7, 12] 1.3 mm [21]). The use of monolithic Y-TZP restorations in crowns necessitates a tooth reduction of 0.5 to 1.5 mm or more to accommodate the ceramic layer thickness [23]. One study [24] reported that the optimal concealing thickness varies depending on the substructure material and that a minimum thickness of 0.8 mm is required for zirconia copings on nickel-chromium alloy abutments. In another study [25], disc-shaped specimens of 0.8 and 1.5 mm thickness were fabricated to assess the impact of yttria content on Y-TZP translucency and masking ability, with results showing that all zirconia specimens effectively masked titanium at a minimum thickness of 1.5 mm. In the present study, as noted above disc-shaped 12 mm in diameter specimens were fabricated with a thickness of 0.8 and 1.5 mm.

Autoclave treatment is frequently used as an accelerated aging test for zirconia ceramics [26]. However, the correlation between autoclaving duration and the aging time measured in vivo remains a controversial issue [27]. It has been reported that 1 h of aging in an autoclave at 134 °C and 2 bar pressure corresponds to 3–4 years of aging in the oral environment [28]. This aging procedure has been applied in various studies with different aging durations [13, 16, 21, 22]. In the present study, the autoclave aging times were equivalent to approximately 3–20 years in a clinical intraoral environment reflecting the estimated lifetime of zirconia service.

Transformation toughening is a process in which stress induces the transformation of tetragonal crystals to monoclinic crystals in stabilized zirconia. This transformation is associated with a significant volume expansion of 3–5%, which generates compressive stresses that prevent crack opening and increase resistance to crack propagation [29].

A study investigated the flexural strength of disc-shaped specimens of four different zirconia materials (3Y-TZP, 4Y-TZP, 5Y-TZP, and 6Y-TZP) after autoclave aging [20]. The results indicated that 3Y-TZP zirconia exhibited higher flexural strength compared to other cubic-containing zirconia materials. It has been stated that higher yttria content stabilizes zirconia with more cubic crystals, eliminating the transformation toughening mechanism [30]. This result can be attributed to the more stable phase of cubic zirconia, which reduces stress-induced transformation toughening [31]. In alignment with the findings of the previous study [20], the average flexural strength in the 5Y groups examined in the present study was below 500 MPa. Consequently, it is recommended that full-contour crowns and three-unit fixed prostheses that do not involve molar restorations be used. However, the (3 + 5)Y group exhibited a greater amount of tetragonal structure due to transformation toughening phenomenon. As a result, the flexural strength for (3 + 5)Y group was greater than 500 MPa but did not exceed 800 MPa. Therefore, it is suitable for fabricating prostheses involving molar restorations with a span of no more than four units, and multi-unit bridges with a maximum of two pontics. Conversely, in the present study, the 3Y groups demonstrated the highest strength. Thus, 3Y is recommended for the fabrication of prostheses involving partially or fully covered substructure for crowns and bridges of any span [32]. However, in [20] there was a partial discrepancy between the results, as the flexural strength of 3Y-TZP decreased with aging. In contrast, the present study observed that the 3Y control group had lower flexural strength values compared to the 3Y group aged for 5 h, a result which may be attributed to the transformation toughening mechanism [29].

Various in vitro studies have demonstrated that the mechanical properties of 5Y-PSZ ceramics are inferior to those of 3Y-TZP and 4Y-PSZ ceramics [30, 33, 34]. One study [6] reported that zirconia with 5.5% and greater than 6% mol Y_2_O_3_ content were resistant to hydrothermal aging for up to 54 h. The results of the present study support these findings [6].

In another study [35], it was found that an increase in the thickness of monolithic zirconia leads to an increase in its flexural strength. Yet, another study [14] reported that both thickness and the flexural strength of the material increased simultaneously, while prolonged aging time resulted in decreased flexural strength. Our findings showed a statistically significant difference in the flexural strength between the two specimen thicknesses, with the mean flexural strength of the 1.5 mm specimens being significantly higher. These results align with those reported in the aforementioned studies [14, 35]. However, unlike one of the previous studies [14], no significant difference was observed between aging times in terms of flexural strength values. The interaction between material type and aging has statistically significant effect only in the case of the 3Y groups. Specifically, the 3Y control group exhibited lower flexural strength values compared to the 3Y-5 group, which may be attributed to the transformation toughening mechanism. Also the results of another study [21] support the present study’s result. The discrepancy between the present study and the previous study [14] regarding the decrease in flexural strength over time, could be attributed to the shorter aging time, in the present study.

The interaction between material type and thickness has a statistically significant effect on flexural strength in the (3 + 5)Y and (4 + 5)Y groups, with flexural strength increasing with thickness in these groups. However, this interaction is not observed in other groups. Although the flexural strength of the 5Y-0.8 group was higher than the 5Y-1.5 group, this difference was not statistically significant. In another study [34] it was concluded that increasing the 5Y-TZP zirconia crown thickness resulted in a moderate but statistically insignificant increase in fracture force. These findings are in line with the present study’s results. According to the manufacturer’s instructions, the required minimum thickness for (3 + 5)Y and (4 + 5)Y materials is 0.8 mm in the anterior region and 1.0 mm in the posterior region. The results of our study support these guidelines. In the posterior region, where greater forces are applied, material thickness becomes crucial. For areas subjected to high forces (such as posterior crowns, long span bridges, and patients with bruxism), it is recommended to increase the thickness of these materials.

Over the years, studies [20–22] assessing the amount of transformation and the biaxial flexural strength have presented conflicting results. This discrepancies are attributed to the varying parameters used in aging. Some studies did not observe any significant impact on the phase transformation after up to 5 h aging in an autoclave [36–38], resulting in nearly 25.4% monoclinic phase at the surface [37].

One study [39] reported that the volume fraction change of the monoclinic phase depends on the brands of translucent zirconia after hydrothermal treatment. Another study [40] proposed that using the equation (19) may lead to inaccurate estimations of monoclinic phase fraction (Fm) in zirconia material containing a significant amount of cubic phase. It was suggested that X-ray diffraction (XRD) analyses can enhance the accuracy of phase composition determinations. However, since XRD analysis was performed on a single specimen from each subgroup in the present study, it is not possible to make a statistical evaluation in terms of flexural strength results. The highest Xm values were obtained in the 3Y-0.8-5 (0.20%), 3Y-1.5-5 (0.17%), (3 + 5)Y-0.8-5 (0.18%), (3Y + 5)-1.5-5 (0.13%) groups. These discrepancies in Xm values may be related to tetragonal-to-monoclinic phase transformation in materials with 3 mol% yttria content. Additionally, the 3Y and (3 + 5)Y groups exhibited higher flexural strength values compared to the other groups. When the Xm values are interpreted alongside the flexural strength results, it can be inferred that transformation toughening likely occurred in these groups, whereas it was not observed in others.

According to the SEM analysis conducted in the present study, the control groups exhibited shallower and, more uniform surfaces, whereas the test groups with extended aging showed greater surface degradation. The susceptibility to low-temperature degradation of zirconia ceramics used in the oral cavity may vary, and this factor should be considered in the application of monolithic zirconia materials. The clinical use of translucent zirconia is important, as indicated by the literature, as materials with high cubic phase and yttria content are more stable.

A limitation of the present study was the use of disc-shaped specimens, which do not mimic the geometry of crown-bridge restorations. However, designing and producing these specimens from the middle of monolithic color and strength-gradient materials provides an advantage in that it mimics the color and strength-gradient properties of crown-bridge restorations. XRD analysis was used to examine the phase transformation in this study. For a more comprehensive understanding of the phase transformation mechanism and in order to evaluate the cubic phase content of highly translucent zirconia materials, it is recommended that detailed phase analysis Raman spectroscopy or Rietveld analysis and refinement be performed. Multi-layered discs possess a complex multi-compositional structure, making it challenging to pinpoint the exact location of phase transformation, given that did not occur uniformly across all layers.

Since this study was conducted in vitro, artificial aging conditions cannot fully replicate the oral environment. Therefore, it is difficult to establish a linear relationship between the results of these studies and the longevity of restorations.

## Conclusions

Within the limitations of this study, it was concluded that:

1. Flexural strength varies depending on the type of zirconia material used, with 3Y-TZP LA (DD Bio ZX^2^ Color) exhibiting the highest flexural strength values among those examined.
2. The thickness of the zirconia material significantly influences its flexural strength.
3. Aging time does not affect the flexural strength values.

Material selection and thickness are crucial factors that clinicians must consider. However, the aging time did not significantly affect the zirconia samples evaluated in this study.

## Data Availability

All data generated or analyzed during this study are included in this published article.
